# Controlled Release of Insulin Based on Temperature and Glucose Dual Responsive Biomicrocapsules

**DOI:** 10.3390/molecules27051686

**Published:** 2022-03-04

**Authors:** Xiaoguang Fan, Shiya Gu, Jingsheng Lei, Shiyan Gu, Lei Yang

**Affiliations:** 1 College of Engineering, Shenyang Agricultural University, Shenyang 110866, China; xiaoguangfan1982@syau.edu.cn (X.F.); gsy1424167584@163.com (S.G.); ljs13317228919@163.com (J.L.); 2 School of Petrochemical Engineering, Liaoning Petrochemical University, Fushun 113001, China

**Keywords:** poly(*N*-isopropylacrylamide), insulin delivery system, copolymer microcapsule, insulinoma β-TC6 cells, fluidized bed reactor

## Abstract

The treatment of diabetes lies in developing novel functional carriers, which are expected to have the unique capability of monitoring blood glucose levels continuously and dispensing insulin correctly and timely. Hence, this study is proposing to create a smart self-regulated insulin delivery system according to changes in glucose concentration. Temperature and glucose dual responsive copolymer microcapsules bearing *N*-isopropylacrylamide and 3-acrylamidophenylboronic acid as main components were developed by bottom-spray coating technology and template method. The insulinoma β-TC6 cells were trapped in the copolymer microcapsules by use of temperature sensitivity, and then growth, proliferation, and glucose-responsive insulin secretion of microencapsulated cells were successively monitored. The copolymer microcapsules showed favorable structural stability and good biocompatibility against β-TC6 cells. Compared with free cells, the biomicrocapsules presented a more effective and safer glucose-dependent insulin release behavior. The bioactivity of secreted and released insulin did not differ between free and encapsulated β-TC6 cells. The results demonstrated that the copolymer microcapsules had a positive effect on real-time sensing of glucose and precise controlled release of insulin. The intelligent drug delivery system is supposed to mimic insulin secretion in a physiological manner, and further provide new perspectives and technical support for the development of artificial pancreas.

## 1. Introduction

In recent years, many researchers have studied multi-responsive materials, especially for functional carries with dual sensitivity to temperature and glucose, and gradually carried out the investigations on intelligent regulation of controlled release of insulin [1,2,3]. Kataoka’s group made the most groundbreaking work in the synthesis, characterization, and application of poly(*N*-isopropylacrylamide) (PNIPAAm) and phenylboronic acid (PBA) as temperature and glucose dual-responsive materials. Their results on glucose-stimulated insulin release obviously showed that the hydrogels shrunk and the amount of insulin released from the hydrogels was small with lower ambient glucose concentration; when glucose concentration reached a certain threshold, insulin escaped significantly from the hydrogels, i.e., the elaborate hydrogels could be structurally altered in response to glucose concentration, successfully implementing the “on/off” control of insulin release [4,5]. Therefore, their work has become the main reference for related follow-up studies.

The studies suggest that if intelligent insulin delivery systems are to be applied to clinical treatment, the carriers have got to simultaneously meet the requirements of excellent biocompatibility, small size, good stability, fast response, strong controlled release capacity, high loading efficiency, and durable repeated switching level, and must also be able to maintain the bioactivity and stability of loaded and released insulin. Although microgels, self-assembled micelles, core–shell microgels, and hollow microcapsules with PNIPAAm and PBA as functional architecture have the potential applications to be used in vivo [6,7,8,9], they have respective shortcomings, such as complex and expensive procedure, small drug loading, poor stability, and hard batch production. Therefore, it is necessary to develop a convenient and economical preparation process for industrialization, reduce overall scale and increase loading space of carriers as much as possible, introduce safe and harmless functional raw/auxiliary materials, and further improve the comprehensive performance of intelligent carriers. This will facilitate the promotion and application of intelligent insulin delivery systems in the treatment of diabetes, such as subcutaneous embedding, intravenous injection, and oral delivery.

Comparing the comprehensive performance of various glucose responsive carriers, microcapsules have remarkable advantages due to the characteristics of small space and large capacity [10,11,12]. However, it is difficult to achieve large-scale production in the existing scheme, except by relying on advanced technology and engineering equipment with increased production capacity. Furthermore, the drug loading capacity of microcapsules is greatly increased but remains limited, so the exogenous insulin trapped inside cannot be released indefinitely. If insulin-producing cells can be packed into microcapsules, a steady supply of insulin will be guaranteed, which can provide long-term glucose-regulated insulin delivery [13,14,15]. The encapsulation systems can prevent cell leakage, protect encapsulated cells from immune attacks, as well as allow the diffusion of nutrients and the secretion of insulin [16,17,18]. 

This study aims to develop a novel strategy for massive preparation of temperature and glucose dual-responsive copolymer microcapsules. Based on the synthetic strategy of thermoresponsive copolymers in the previous studies [19,20], the glucose-sensitive PBA groups were introduced into the molecular chains of PNIPAAm, and then the preparation procedure of temperature-sensitive copolymer films was also used in this study. The copolymer solution was uniformly coated on the surfaces of microspheres to generate core–shell microspheres by using bottom-spray fluidized bed technology, and the microsphere templates were removed to form copolymer microcapsules. So far, little information is available about the fabrication of core–shell microspheres (transitional form of microcapsules) by means of fluidized bed reactors. This process is expected to accomplish large-scale preparation of functional microcapsules and provide a basis for further clinical applications of intelligent insulin delivery systems. On this basis, insulinoma β-TC6 cells will be encapsulated in temperature and glucose dual-responsive copolymer microcapsules. When glucose is introduced into the hybrid systems, the materials and cells will respond to glucose successively. The bonding of microcapsules and glucose causes the base materials to swell, and further β-TC6 cells to secrete high-quality insulin under glucose stimulation. This pattern can not only provide safe and stable immune barriers for β-TC6 cells, but also closely simulate the microenvironment for cell growth, proliferation, and functionalization, which can offer reliable sources of insulin. Meanwhile, this study develops novel intelligent glucose-responsive biomicrocapsules, whose duration of drug release is approximately equal to the average life span of cells (more than 50 passages, over a year) [13,21,22,23] in theory. It is beneficial to reduce treatment times so as to improve the medication compliance of diabetes patients, and contributes to the development of new artificial pancreas.

In this study, the thermo-glucose dual-responsive copolymer microcapsules were prepared by spray coating, thermal annealing, and template removal, and the chemical composition, swelling ratio, surface topology, and structure stability were further investigated. Encapsulation, growth, and glucose-responsive insulin secretion of β-TC6 cells in the copolymer microcapsules were monitored successively. The bioactivity of secreted and released insulin derived from microencapsulated β-TC6 cells was also determined.

## 2. Results and Discussion

### 2.1. Preparation of Temperature and Glucose Dual-Responsive Copolymer Microcapsules

**Figure 1:**
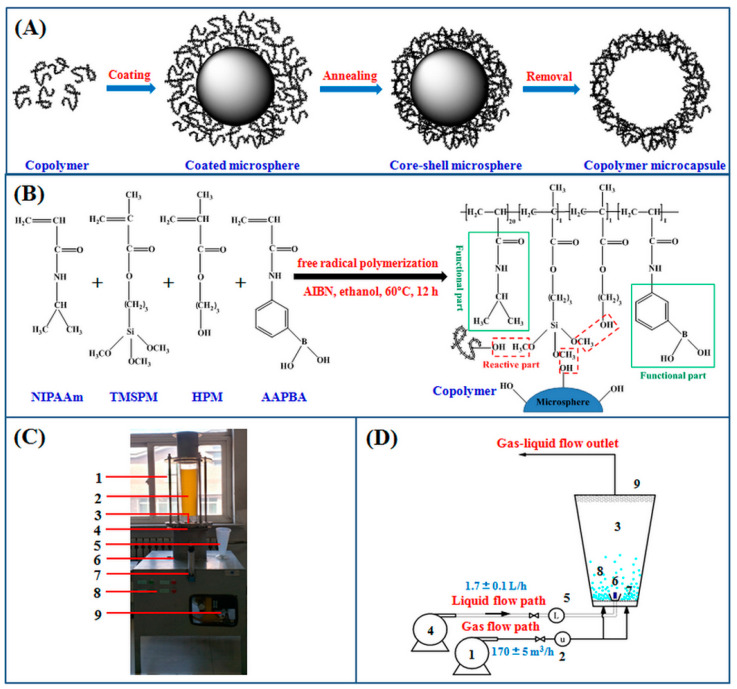
(**A**) Schematic diagram of copolymer microcapsule formation. (**B**) Schematic illustration for the synthesis of p(*N*-isopropylacrylamide-*co*-3-acrylamidophenylboronic acid-*co*- hydroxypropyl methacrylate-*co*-3-trimethoxysilylpropyl methacrylate), p(NIPAAm-*co*-AAPBA-*co*-HPM-*co*-TMSPM) copolymers, and the formation of copolymer film with clear indication of three possible types of methanol removal when annealed. (**C**) Experimental rig of bottom-spray fluidized bed reactor system: 1 fixed support, 2 main reactor, 3 atomizing nozzle, 4 air distribution plate, 5 reservoir, 6 air inlet chamber, 7 volume flowmeter, 8 control device, 9 auxiliary equipment. (**D**) Flow diagram of bottom-spray coating technology: 1 fan, 2 anemometer, 3 bottom-spray fluidized bed reactor, 4 diaphragm metering pump, 5 volume flowmeter, 6 atomizing nozzle, 7 air distribution plate, 8 glass mirospheres, 9 superfine mesh.

This study provided a useful and effective route for the development of thermo-glucose dual-responsive copolymer microcapsules to facilitate cell encapsulation. As shown in Figure 1A, p(*N*-isopropylacrylamide-*co*-3-acrylamidophenylboronic acid-*co*-hydroxypropyl methacrylate-*co*-3-trimethoxysilylpropyl methacrylate), p(NIPAAm-*co*-AAPBA-*co*-HPM-*co*-TMSPM) copolymers were first synthesized by free radical polymerization, and then the copolymer solution was evenly coated on the surfaces of glass microspheres through bottom-spray coating technology. Subsequently, the core–shell microspheres were availably fabricated by thermal annealing, and the microcapsules were finally formed by removing the microspheres via hydrofluoric acid (HF) solution. 

P(NIPAAm-*co*-AAPBA-*co*-HPM-*co*-TMSPM) copolymers were prepared from the monomers of *N*-isopropylacrylamide (NIPAAm), 3-acrylamidophenylboronic acid (AAPBA), hydroxypropyl methacrylate (HPM), and 3-trimethoxysilylpropyl methacrylate (TMSPM) with final molar ratios of nearly 20:1:1:1. The number-average molecular weight and weight-average molecular weight of the copolymer nanoparticles were 1.5 × 10^4^ g∙mol^−1^ and 2.4 × 10^4^ g∙mol^−1^, respectively. The lower critical solution temperature (LCST) of the copolymers in deionized water was about 23 °C, and the p*K*_a_ was around 7.28. As illustrated in Figure 1B, NIPAAm and AAPBA used as primary reactants provided their functional groups, which enabled the copolymers to have temperature and glucose responses. The siloxane bonds derived from TMSPM could couple with hydroxyl containing substrates with any shape and dimension by heating or radiation [24,25]. In addition, HPM could give their effective hydroxyl groups for the intermolecular bonding of temperature and glucose dual-responsive copolymers. Therefore, the elaborate copolymer nanoparticles were capable of depositing onto glass microspheres to form network architecture. The synthesis of copolymers with smart responses was the precondition and foundation for subsequent preparation of functional microcapsules. 

The preparation scheme of core–shell microspheres (transitional form of microcapsules) was designed based on the previous production strategy of temperature sensitive copolymer films formed on glass coverslips [19]. However, the size and shape of the glass microspheres were markedly different from those of two-dimensional substrates, thus the traditional making techniques for planar membranes were inapplicable to control the grafting uniformity and film thickness. The bottom-spray fluidized bed technology has been widely used to obtain homogeneous and dense films on particle surfaces [26,27,28]. Therefore, a self-made bottom-spray fluidized bed reactor [29] (see Figure 1C,D) was introduced in this study, by taking advantage of distribution uniformity and easy control to achieve a uniform coating and thickness accumulation on the glass microspheres. The main body of the fluidized bed reactor was an inverted cone with an upper bottom diameter of 13 cm, lower bottom diameter of 8 cm, height of 78 cm, and cone angle of 10°. The reactor was equipped with ultrafine mesh at the top and an air distribution plate at the bottom. The pores of the distribution plate were evenly distributed with an aperture of 5 mm and a spacing of 8 mm. To our limited knowledge, there were no studies on the fabrication of smart responsive composite carriers by virtue of a fluidized bed reactor. This strategy was expected to accomplish a predictable and cost-effective scaling up of smart composite carriers, to bring benefits to industrial application and popularization.

As mentioned above, the uniform and unlinked coatings were formed on the microspheres with the help of a fluidized bed reactor. Covalent crosslinking among copolymers and templates was then constructed by a methanol removal reaction between siloxane bonds and hydroxyl groups by heating to 125 °C for 3 h under vacuum. Hence, the core–shell microspheres with glass core and multi-responsive shell were produced successfully. The glass templates of composite particles were often removed using HF solution under certain conditions to produce functional microcapsules [30,31,32]. In this study, therefore, the temperature and glucose dual-responsive copolymer microcapsules were obtained by exposure to HF solution.

### 2.2. Characterization of Temperature and Glucose Dual Responsive Copolymer Microcapsules

#### 2.2.1. Component Analysis

**Figure 2:**
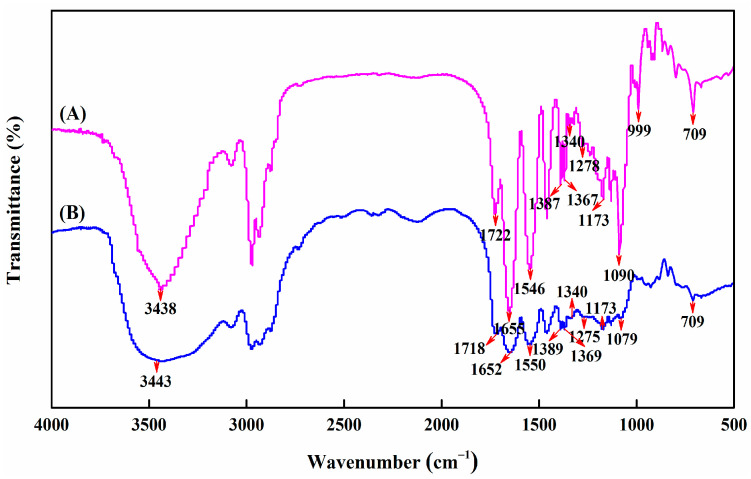
Attenuated total reflection–Fourier transform infrared (ATR–FTIR) spectra of copolymer microcapsules (A) and nanoparticles (B).

Figure 2 shows the comparison of attenuated total reflection–Fourier transform infrared (ATR-FTIR) spectra for p(NIPAAm-*co*-AAPBA-*co*-HPM-*co*-TMSPM) copolymer microcapsules and nanoparticles. Herein, the copolymer nanoparticles were defined as the copolymers synthesized by free radical polymerization, and then purified and lyophilized. The copolymer microcapsules referred to the products after successive treatments of spray coating, thermal annealing, and template etching. The copolymer microcapsules were made from copolymer nanoparticles used as precursors. Primarily, the characteristics peaks at around 1630 cm^−1^ (C=C), believed to represent double-bonded monomers [33,34,35], were not present in both spectra. This demonstrated the successful synthesis of p(NIPAAm-*co*-AAPBA-*co*-HPM-*co*-TMSPM) copolymers via free radical polymerization. Similarly to the results of copolymer nanoparticles, the stretching vibration absorption peak of amide bond I (C=O), and the bending vibration absorption peak of amide bond II (N-H) appeared at 1655 cm^−1^ and 1546 cm^−1^, respectively, the symmetric deformation vibration absorption peaks of isopropyl groups displayed at 1387 cm^−1^ and 1367 cm^−1^ [6], and the characteristic absorption peak of borate groups was observed at 1340 cm^−1^, and the out-of-plane bending vibration absorption peak of benzene ring was detected at 709 cm^−1^ [36]. This suggested that the copolymer microcapsules retained NIPAAm and AAPBA components. Differently, the characteristic absorption peaks of hydroxyl groups (3100~3700 cm^−1^) within the copolymer microcapsules became markedly narrow, and the characteristic absorption peak of siloxane bonds (1079 cm^−1^) was significantly redshifted. These newly emerged strong bands at 1090 cm^−1^ and 999 cm^−1^ were attributed to Si-O and Si-O-Si, respectively [37,38]. This indicated that the molecular chains of copolymer microcapsules were interlinked by crosslinking of hydroxyl and 3-trimethoxysilyl groups, and also confirmed that the removal of microsphere templates did not result in the rupture and separation of copolymer microcapsules. 

To summarize, the isopropyl groups and amide bonds representing thermosensitive functional groups, as well as phenylboric acid groups indicating sugar-sensitive functional groups, still presented in the chemical structure of microcapsules, which was the key evidence that the elaborate microcapsules had dual-responsive properties. Furthermore, new Si-O and Si-O-Si linkage were produced by the methanol removal reaction between siloxane bonds from TMSPM and hydroxyl groups from HPM and substrates to form a copolymer network, resulting in the disappearance of the characteristic peaks of siloxane bonds and hydroxyl groups in the spectrum of copolymer microcapsules.

#### 2.2.2. Equilibrium Swelling Analysis

Whether the microcapsules, after multiple treatments, maintained temperature and glucose dual-responsive characteristics derived from copolymer nanoparticles, was one of the key issues investigated in this study. In order to avoid larger experimental errors caused by water entering into the hollow spaces of microcapsules, the core–shell microspheres, as alternatives to copolymer microcapsules, were used for equilibrium swelling measurements. Although the swelling and shrinkage of the shell walls of core–shell microspheres were affected by the “glass cores” to some extent, the response characteristics of copolymer microcapsules and core–shell microspheres would not essentially change. 

**Figure 3:**
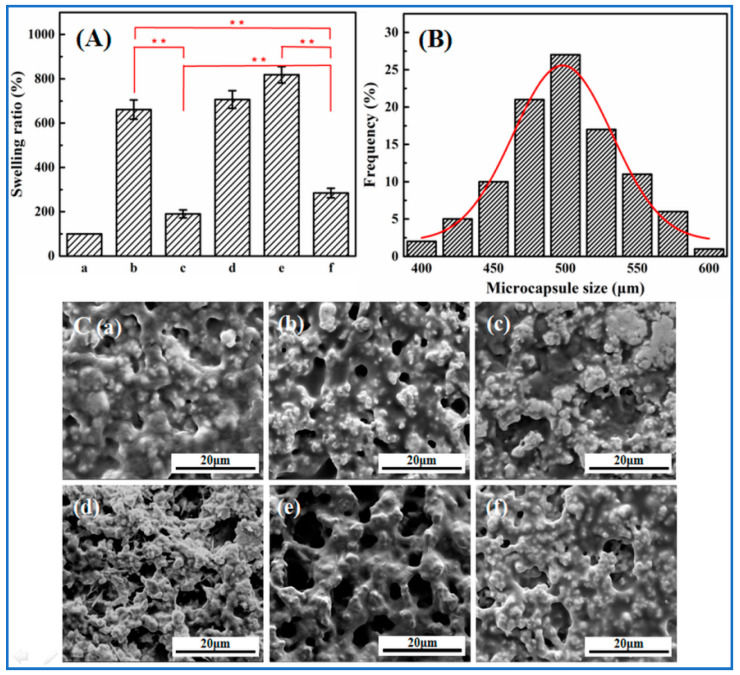
Swelling ratio (**A**), size distribution (**B**), and surface topology (**C**) of copolymer microcapsules in different solutions. (**a**) Dry state; (**b**) deionized water at 20 °C; (**c**) deionized water at 40 °C; (**d**) phosphate buffered solution (PBS) at 20 °C, pH 7.4; (**e**) 5.0 mg∙mL^−1^ glucose solution at 20 °C, pH 7.4; (**f**) 5.0 mg∙mL^−1^ glucose solution at 40 °C, pH 7.4. ** *p* < 0.05 is considered to be statistically significant.

**Figure 4:**
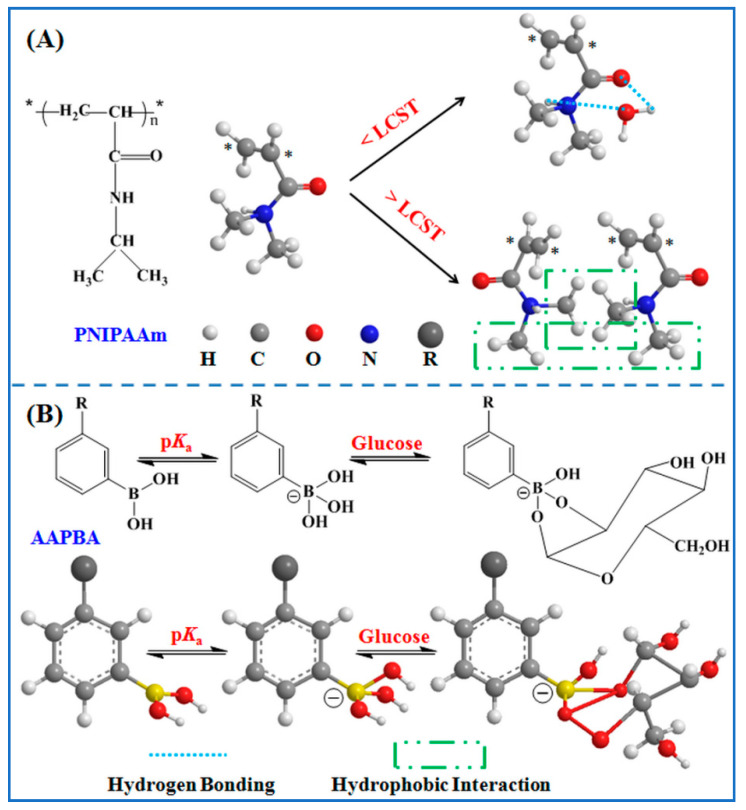
Poly(*N*-isopropylacrylamide) (PNIPAAm) and its mechanism of thermosensitivity (**A**), phenylboronic acid (PBA) and its mechanism of sugar-sensitivity (**B**).

Figure 3A shows the swelling ratios of core–shell microspheres in deionized water at 20 °C (lower than LCST) and 40 °C (higher than LCST), PBS solution with pH 7.4 at 20 °C, as well as 5.0 mg∙mL^−1^ glucose solution with pH 7.4 at 20 °C and 40 °C. Generally, the swelling ratios of all modified microspheres increased to the maximum rapidly and reached swelling equilibrium within 30 min. The swelling ratio of core–shell microspheres in deionized water at 20 °C was about 660%, but dropped to around 190% at 40 °C. This observation shows the copolymer shells had temperature-stimulated responses. Upon the immersion at 20 °C, water quickly filled the copolymer shells of modified microspheres, possibly due to their open structural network. The water molecules bound with amide groups through hydrogen bonding, leading to larger swelling ratio. When heating to 40 °C, hydrophobic interactions between isopropyl groups played dominant roles in copolymer systems, so the swelling ratio markedly decreased, as illustrated in Figure 4A. At the same temperature, the swelling ratio of core–shell microspheres in PBS solution increased slightly, but the difference was not significant compared with that in deionized water. 

In glucose solution, a substantial increase in the swelling degree of modified microspheres was found at both 20 °C (about 820%) and 40 °C (nearly 280%), compared to the results in deionized water at applied temperatures. This phenomenon could be attributed to the fact that phenylboronic acid groups would be converted into hydrophilic and negatively-charged phenyl borate esters in the presence of glucose. The electrostatic repulsion and hydrated hydrogen bonds led to the expansion of the polymeric network, which attracted more water into the network of copolymer shells, as displayed in Figure 4B. The results clearly indicated that the prepared core–shell microspheres exhibited a sugar-sensitive characteristic.

#### 2.2.3. Structural Analysis

The sizes of near-spherical p(NIPAAm-*co*-AAPBA-*co*-HPM-*co*-TMSPM) copolymer microcapsules were determined by electronic micrometer. The produced microcapsules were in the size range of 400~600 µm and followed the Gaussian distribution in which the maximum number of microcapsules were of the size 500 µm, as shown in Figure 3B. In general, the microcapsule diameter would increase with the increase in template size, solution concentration, and spraying time. 

Scanning electron microscopy (SEM) was also applied to examine the changes of surface structure of the copolymer microcapsules to illustrate their temperature-sensitivity and sugar-sensitivity. Figure 3C exhibited the surface topology of the copolymer microcapsules with the diameter of about 500 µm upon immersing in different aqueous solutions followed by isothermal vacuum drying. It could be seen that compared with the copolymer microcapsules prepared by direct drying, the surface topology of the copolymer microcapsules soaked in various aqueous solutions changed significantly. This was caused by water molecules entering into the pore structure of the microcapsules. In deionized water at 20 °C, the surface of the copolymer microcapsule was porous, whereas the mesopores almost disappeared when the temperature rose to 40 °C, which was due to the changes of molecular chains from hydrophilic to hydrophobic and the overall structure from loose to compact at temperatures higher than LCST. This result confirmed that the copolymer microcapsules had temperature-sensitive responses. 

In the phosphate buffered solution (PBS) solution with pH 7.4 at 20 °C, the surface of the copolymer microcapsule was coarse and poriferous, whereas the size and number of pores on the surface of microcapsule increased notably in the glucose solution with the concentration of 5.0 mg·mL^−1^ at 20 °C. In 5.0 mg·mL^−1^ glucose solution at 40 °C and pH 7.4, the pore size and number of the copolymer microcapsule also increased, but not as obvious as it would be at 20 °C under other equal conditions. The surfaces of the microcapsules soaked in glucose solution under pH 7.4 at 37 °C also showed similar results. This suggested that the internal structure of copolymer microcapsules was adjusted under the stimulation of glucose, i.e., the copolymer microcapsules had intelligent sensing of glucose. As explained by the increased swelling ratio of microcapsules in the presence of glucose, the charged phenylboronic acid groups could bind glucose to produce reversible covalent complexes that increased the amount of negative charge of PBA, thus forming a more hydrophilic structure. The variations in pore size and number were sufficient to control insulin release, learning from the results of glucose-responsive secretion and release of insulin. Therefore, the copolymer microcapsules have attractive potentials as new tools for controlled release of insulin under physiological conditions.

#### 2.2.4. Stability Analysis

**Figure 5:**
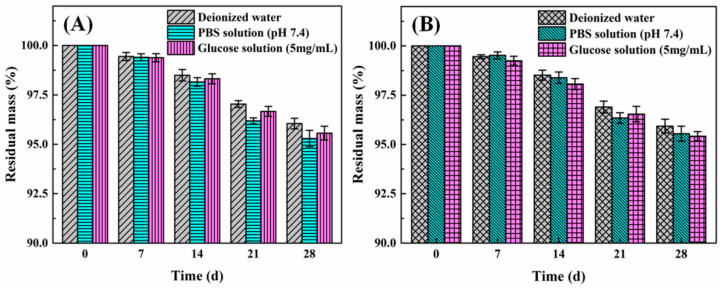
Degradation of copolymer microcapsules in different solutions including deionized water, phosphate buffered solution (PBS) with pH 7.4 as well as 5.0 mg∙mL^−1^ glucose solution under 20 °C (**A**) and 40 °C (**B**) at regular intervals.

The stability of drug delivery carriers is an important factor in in vivo application, which can effectively reduce administration times of patients. Figure 5 shows the degradation of the copolymer microcapsules with the diameter of around 500 µm. It can be seen that the residual mass of the copolymer microcapsules remained more than 95% although they were soaked in different solutions for up to 28 days either at 20 °C or 40 °C. Furthermore, there was no significant difference in the results among the three solutions including deionized water, PBS solution (pH 7.4), and 5.0 mg∙mL^−1^ glucose solution at both temperatures on the same testing day. The mass loss might be caused by insufficient crosslinking of a small amount of copolymer molecular chains [39,40]. The finding was enough to demonstrate the excellent stability of the selected copolymer microcapsules [14,18]. Similar experimental results were obtained for other copolymer microcapsules with different diameters. In the present work, thermal annealing was found to improve the stability of copolymer microcapsules, because this treatment facilitated the crosslinking of hydroxyl and 3-trimethoxysilyl groups to generate a network structure for microcapsule walls.

### 2.3. Encapsulation and Growth of β-TC6 Cells

β-TC cell line is derived from insulinoma cells of transgenic mice expressing SV40 T antigen under the control of an insulin promoter, which can retain stable differentiation for about 50 passages in in vitro culture and has appropriate glucose-induced insulin responsivity within the range between 5 and 30 mM for glucose concentration [13,21,22,23]. This cell line has been proved to have remarkably similar characteristics to β-cells of pancreatic islets, so it can be used to replace normal mouse pancreatic β cells for basic research on diabetes mellitus. Therefore, the insulin-producing β-TC6 cells were selected in this study. 

Three types of culture media were employed in this study: high-sugar medium containing 4.5 mg∙mL^−1^ glucose in solution, low-sugar medium containing 1.0 mg∙mL^−1^ glucose in solution, as well as sugar-free medium with no glucose in solution. High-sugar culture medium is often used for in-vitro cultivation of tumor cells, which is conducive to cell growth and proliferation [14,22,23]. Therefore, in this study, high-sugar culture medium was applied for routine culture of free and microencapsulated β-TC6 cells. Low-glucose culture medium was used for long-term glucose stimulated insulin secretion and release of microencapsulated β-TC6 cells under the same stimulus, and the glucose concentration of 1.0 mg∙mL^−1^ was suitable for simultaneous stimulation of copolymer microcapsules and encapsulated cells. Sugar-free culture medium was used in cell encapsulation, and also used as basal solutions that regulated glucose concentration during glucose stimulation of different concentrations. 

**Figure 6:**
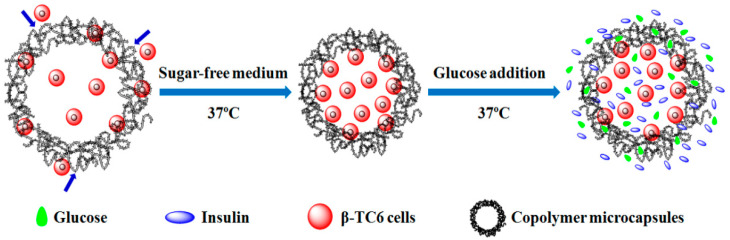
Schematic diagram of encapsulation of β-TC6 cells, and glucose-responsive insulin secretion and release of cells entrapped in copolymer microcapsules.

Firstly, β-TC6 cells were encapsulated by multi-site injection method utilizing the thermosensitive properties and capillary force of copolymer microcapsules, as illustrated in Figure 6. The fully swollen copolymer microcapsules at 20 °C exhibited many pores with different sizes after isothermal vacuum drying. Therefore, β-TC6 cells rapidly entered the interior of the copolymer microcapsules driven by capillary force, when cell suspension with the temperature of 4 °C was injected into the microcapsules carefully. Then, the microcapsules loaded with β-TC6 cells were placed in sugar-free medium at 37 °C. As the temperature increased, the pore size of the microcapsules was rapidly reduced, thus β-TC6 cells were enclosed in the copolymer microcapsules. This approach of cell encapsulation allowed more cells to flow into the microcapsules readily, and the cells had difficulty escaping from the narrowing pores, thus the encapsulation efficiency of β-TC6 cells achieved more than 90% in this study.

**Figure 7:**
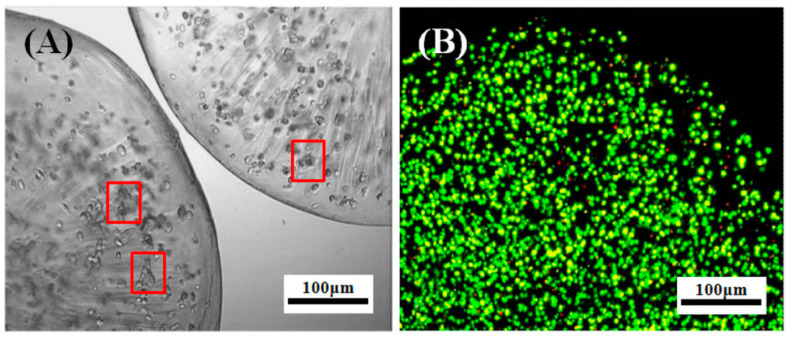
Growth (**A**) and viability (**B**) of β-TC6 cells cultured in copolymer microcapsules on Day 7. The red rectangle marked some representative small clusters of β-TC6 cells.

Cell viability was evaluated by double staining of calcein-acetylmethyl (AM) and propidium iodide (PI) every 24 h after cell seeding. Meanwhile, cell morphology was observed by inverted fluorescence microscope, and the growth and proliferation of β-TC6 cells under encapsulation and conventional culture modes were compared by means of the cell counting kit-8 (CCK-8) method. Figure 7 clearly shows the growth and viability of β-TC6 cells cultured in the copolymer microcapsules on Day 7. The appearance of the copolymer microcapsules was intact, and no obvious suspended cells were observed in the culture medium, indicating that the copolymer microcapsules were tightly structured at 37 °C and β-TC6 cells were unable to escape from the niches. In addition, the scattered cells were observed at the initial stage of incubation into the microcapsules, whereas β-TC6 cells initially formed several small clusters a week later. The dead/alive fluorescence staining images of microencapsulated β-TC6 cells presented that there were only a few necrotic or apoptotic cells (red fluorescence) in the copolymer microcapsules, indicating that after 7 days of static culture in vitro, β-TC6 cells still had good activity (green fluorescence) in the microcapsules. The copolymer microcapsules with the significant narrowing of pores at 37 °C could effectively prevent cell leakage, providing safer and larger niches for cell activity and allowing the diffusion of nutrients and metabolites. Therefore, the microencapsulated β-TC6 cells showed good growth and high viability. Other related studies show similar findings. Chaimov et al. [16] developed an extracellular matrix (ECM) encapsulation platform for human liver cells and mesenchymal stem cells, and they demonstrated that the ECM-microcapsule platform provided a natural fibrous three-dimensional niche and supported cellular viability and differentiation, while significantly improving insulin delivery. Leroux et al. [17] fabricated a hybrid alginate@TiO_2_ microcapsule as a reservoir for rat insulinoma-derived INS-1E cells, and the relults revealed that the encapsulated cells showed higher metabolic activity and maintained insulin secretion over more than 6 weeks. Nikravesh et al. [18] prepared alginate microcapsules loaded with pancreatic β-cells via a vibrating nozzle system and used a fluidized-bed bioreactor for the cultivation of encapsulated cells, and the results confirmed the stability of encapsulated systems under fluidized culture and indicated that the endocrine β-cells with higher viability cultured in the bioreactor were shown to be dramatically more responsive to the changes in glucose concentration compared to static culture.

**Figure 8:**
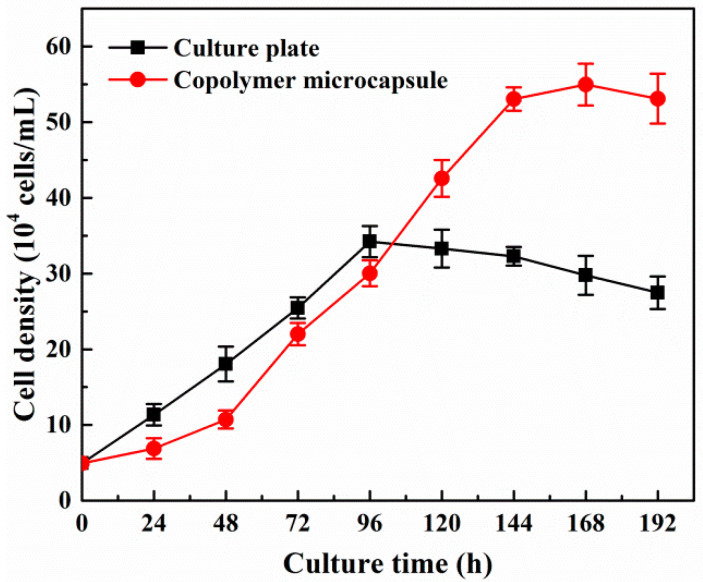
Growth curves of β-TC6 cells in different culture manners.

The growth curves of β-TC6 cells against microencapsulated culture (copolymer microcapsules) and conventional culture (24-well culture plates) are statistically shown in Figure 8. The maximum amplification of β-TC6 cells in different culture manners was calculated according to the ratio of the cell number at the maximum growth peak to the initial stage of cell seeding. It could be found that the proliferation rate of β-TC6 cells in culture plates was relatively faster at the early stage of static culture, because it would take some time for β-TC6 cells to adapt to a new culture environment after seeding in copolymer microcapsules, so the cells achieved the highest amplification rate on Day 4 in the culture plates (6.83 ± 0.92), but on Day 7 in microcapsules (11.07 ± 0.39). However, the maximum amplification of β-TC6 cells cultured in copolymer microcapsules was significantly higher than that of cells grown in culture plates. The cell mass within the microcapsules was observed to expand in a three-dimensional fashion, in contrast to cells seeded on culture plates that grew as a monolayer [13]. The copolymer microcapsules provided cells with a more similar physiological microenvironment, which could effectively overcome cell contact inhibition and was highly conducive to cell expansion, thus remarkably increasing the number of β-TC6 cells.

### 2.4. Glucose-Responsive Secretion and Release of Insulin

As shown in Figure 6, in the presence of glucose (also including 4.5/1.0 mg∙mL^−1^ glucose in high glucose/low glucose-Dulbecco’s modified Eagle’s medium (HG/LG-DMEM)), the copolymer microencapsules and β-TC6 cells would undergo the following changes regardless of temperature above LCST: Firstly, the copolymer microcapsules swelled and the pore sizes became larger, but the increase was not enough to cause cells to escape from them. The glucose that was not bound to the copolymer microcapsules could penetrate the microcapsules successfully through the pores and fully contact with the encapsulated cells, which availably promoted β-TC6 cells to secrete insulin, and the secreted insulin could be discharged out of the microcapsules through the pores. 

After cell encapsulation in copolymer microcapsules, β-TC6 cells were cultured in low-sugar medium at 37 °C, and the used medium was replaced with low-sugar culture medium every 3 days. β-TC6 cells secreted insulin under the stimulation of glucose in the culture medium. After a certain amount of time, the culture medium was collected and the insulin content was determined by mouse insulin enzyme-linked immunosorbent assay (ELISA) kit. Previous studies showed that in static incubations, the glucose concentration at 0.5 mM (i.e., 0.09 mg∙mL^−1^) provided an apparent half-maximal response, and the maximal response was achieved by a glucose concentration of 2.8 mM (i.e., 0.5 mg∙mL^−1^) [41]. However, in this study, exogenous glucose was required to serve double duty. It was used to stimulate the insulin secretion of β-TC6 cells, and facilitate the pore enlargement of temperature and glucose dual responsive copolymer microcapsules, thus the concentration (5.6 mM, i.e., 1.0 mg∙mL^−1^) for glucose stimulated insulin secretion during an extended period was designed to be higher than that used in other studies. 

**Figure 9:**
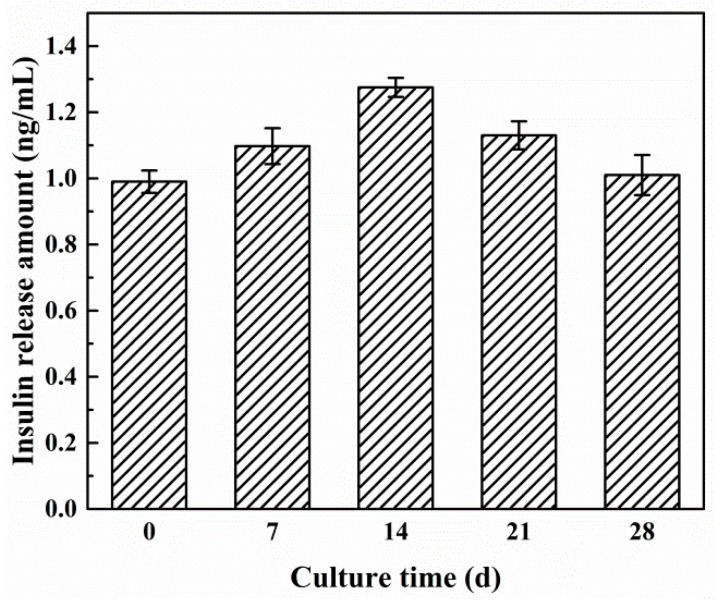
Glucose stimulated insulin secretion and release by microencapsulated β-TC6 cells under the same glucose concentration of 1.0 mg∙mL^−1^ at regular intervals.

The ability of microencapsulated β-TC6 cells to secrete insulin in the presence of glucose (1.0 mg∙mL^−1^ in LG-DMEM medium) over a period of 28 days at fixed intervals is displayed in Figure 9. The results show that β-TC6 cells trapped in the copolymer microcapsules still retain the ability to function efficiently in response to glucose for a relatively longer period of time. It could be found there were some slight differences in the amount of insulin secreted by encapsulated β-TC6 cells during this period. The amount of secreted and released insulin showed a minimum on Day 0 and Day 28, a rising level in the middle days, and then a drop from Day 21. On Day 0, the encapsulated β-TC6 cells were just transferred to a new growth environment, so they could not express fully on glucose stimulation. Under low or no glucose conditions, cell proliferation slowed, but the number of cells increased steadily and modestly along with culture time, thus the amount of secreted and released insulin somewhat increased with a slight increase in the number of cells. Furthermore, a decrease in the secretion of insulin from Day 21 might be owing to the imperfection in the permeability of capsule walls, accordingly affecting the free movement of essential nutrients required for the survival and insulin secreting ability of encapsulated β-TC6 cells [14]. 

**Figure 10:**
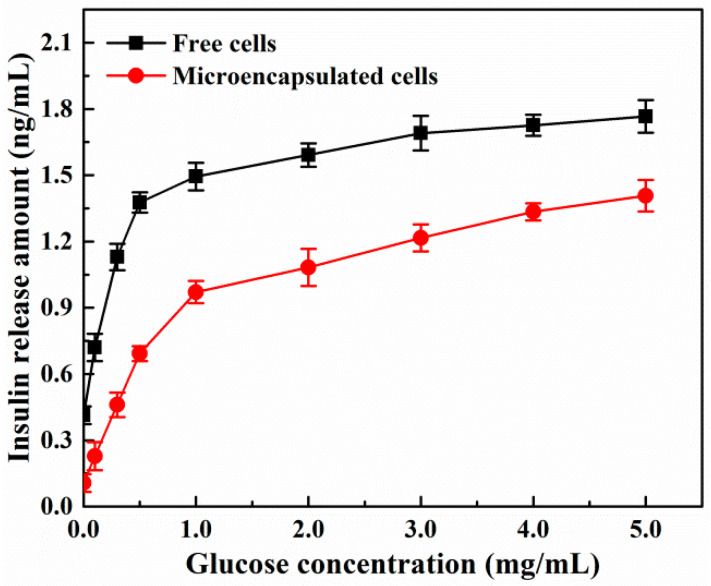
Glucose stimulated insulin secretion and release of β-TC6 cells with gradient increased glucose concentration (0~5.0 mg∙mL^−1^).

The glucose concentration in sugar-free culture medium was gradient increased to determine the secretion and release of insulin at different stimulus concentrations. Figure 10 comparatively illustrated the responses of free and microencapsulated β-TC6 cells to glucose concentration. For free β-TC6 cells, the amount of insulin secretion was the amount of released insulin. In the absence of glucose, free β-TC6 cells still secreted a small amount of insulin (0.41 ± 0.040 ng/mL). The secretion of insulin increased rapidly when the glucose level increased from 0 to 0.5 mg∙mL^−1^, and the insulin secretion (1.38 ± 0.046 ng/mL) at 0.5 mg∙mL^−1^ glucose concentration was almost twice as that (0.72 ± 0.062 ng/mL) at 0.1 mg∙mL^−1^ glucose concentration. When the glucose content gradually increased from 0.5 to 5.0 mg∙mL^−1^, the insulin secretion showed a slight dose-dependent increase. This finding was consistent with the results shown in previous reports [13,41]. For microencapsulated β-TC6 cells, the amount of insulin secretion was not equal to the amount of release due to the shielding effect of copolymer microcapsules [14]. Throughout the range of glucose concentration, the secretion and release of insulin derived from encapsulated β-TC6 cells went up steadily with the increasing glucose content. No or lower glucose content in the solution, such as 0.1 mg∙mL^−1^, was not enough to cause obvious changes in the structure of copolymer microcapsules, although β-TC6 cells had already secreted some insulin which was blocked inside the microcapsules, so lower insulin measurements (0.11 ± 0.040 ng/mL and 0.23 ± 0.064 ng/mL, respectively) were detected. The glucose concentration of 1.0 mg∙mL^−1^ was chosen as a boundary between the two zones, rapid rise and slow rise. When the glucose content exceeded 1.0 mg∙mL^−1^ in solution, the insulin release amount of free cells and encapsulated β-TC6 cells increased slowly and synchronously, but the differences were getting smaller. This was due to the fact that the pore size and number of copolymer microcapsules increased with the rise of glucose concentration, allowing more insulin to be released from the microcapsules. This pattern of insulin release is favorable, because the amount of released insulin is almost at the peak while the higher glucose in solution is being consumed (i.e., decreasing); when the glucose levels are lower, the release of insulin accordingly decreases, which is very similar to the behaviors of insulin secretion and release in human body. Therefore, the biomicrocapsules developed in this study are very effective for clinical treatment of diabetes.

### 2.5. Bioactivity of Released Insulin

**Figure 11:**
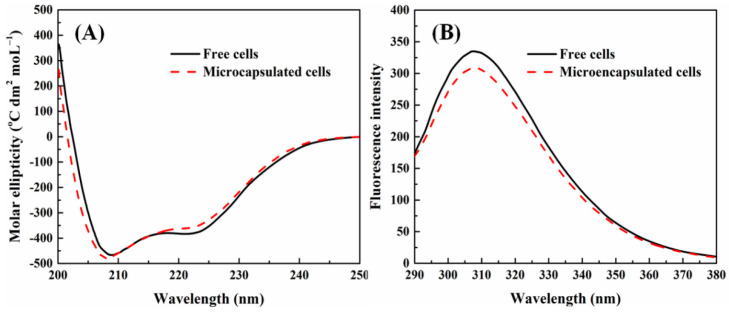
Circular dichroism spectra (**A**) and fluorescence emission spectra (**B**) of released insulin derived from free and microencapsulated β-TC6 cells.

The studies show that after denaturation, not only was the original tightly ordered structure of proteins changed into a disordered and loose extensional chain structure, but also the α-helix structure in the secondary structure was transformed into a β-sheet structure to some extent [42,43]. Figure 11A exhibited the circular dichroism spectra of released insulin derived from free and microencapsulated β-TC6 cells. The ellipticity troughs were observed at 208 nm and 222 nm in both cases, corresponding to the characteristic peaks of α-helical structure of proteins, in accordance with the previous reports [44,45]. The circular dichroism spectrum of insulin released from the biomicrocapsules was very close to that of insulin secreted by free β-TC6 cells, inferring that the matrix materials and treatment procedure did not significantly influence the secondary structure of secreted and released insulin. 

Fluorescence measurements can give information about the molecular environment in the vicinity of the chromophore molecules [45]. Insulin contains four tyrosine (Tyr) residues, three phenylalanine (Phe) residues, and no tryptophan (Trp) residues. When the excitation wavelength is set at 276 nm, only Tyr emits fluorescence, and the emission from Phe can be neglected [45,46]. Therefore, fluorescence emission spectra were also used to characterize and compare the structural stability of secreted and released insulin by free and encapsulated β-TC6 cells. As shown in Figure 11B, the maximum emission wavelength of released insulin in both cases all appeared at 307~309 nm with the excitation wavelength of 276 nm, indicating that there were no specific and obvious shifts in the peak position. The result confirmed that the conformation of insulin released from the biomicrocapsules was not damaged, and it was also verified indirectly that the released insulin remained bioactive [45]. Combining the characterization results of the two spectra, it was indicated that the secondary and tertiary structures of secreted and released insulin did not significantly change through the processes of controlled release. This was mainly due to the reasonable use of the thermosensitive and glucose-sensitive characteristics of the copolymer microcapsules.

## 3. Materials and Methods

### 3.1. Materials and Instruments

P(*N*-isopropylacrylamide-*co*-3-acrylamidophenylboronic acid-*co*-hydroxypropyl methacrylate-*co*-3-trimethoxysilylpropyl methacrylate), p(NIPAAm-*co*-AAPBA-*co*-HPM -*co*-TMSPM) copolymers with the molar ratio of monomers of 20:1:1:1, self-made [47], and all monomers and initiator 2,2-azobisisobutyronitrile (AIBN) were purchased from Sigma-Aldrich, St. Louis, MO, USA; glucose, PBS (pH 7.4), high glucose/low glucose-Dulbecco’s modified Eagle’s medium (HG/LG-DMEM), DMEM, penicillin/streptomycin (P/S), fetal bovine serum (FBS), trypsin, ethylene diamine tetraacetic acid (EDTA), glass microspheres (425~600 μm), Sigma-Aldrich, St. Louis, MO, USA; Insulinoma β-TC6 cells, ATCC, Manassas, VA, USA; cell counting kit-8 (CCK-8), calcein-acetylmethyl (AM)/propidium iodide (PI) double staining kit, Dojindo, Kumamoto, Kyushu, Japan; mouse insulin enzyme-linked immunosorbent assay (ELISA) kit, Millipore, St. Louis, MO, USA. 

Attenuated total reflection–Fourier transform infrared spectrometer (ATR-FTIR, Bruker TENSOR27), BRUKER OPTIK GmbH, Ettlingen, Baden-Wurttemberg, Germany; scanning electron microscope (SEM, Quanta 400 FEG), FEI, Hillsboro, OR, USA; inverted phase contrast fluorescence microscope (TE2000-U), Nikon, Tokyo, Japan; fluorescence spectrometer (F-4500), Hitachi, Tokyo, Japan; circular dichroism spectrometer (J180), JASCO, Tokyo, Japan; Colorimetric microplate reader (ELX800TM), Bio-Tek, Winooski, VT, USA; gas–liquid–solid three-phase bottom-spray fluidized bed reactor, self-made [29].

### 3.2. Preparation and Characterization of Thermo-Glucose Dual-Responsive Copolymer Microcapsules

P(NIPAAm-*co*-AAPBA-*co*-HPM-*co*-TMSPM) copolymers were synthesized by free radical polymerization of NIPAAm, AAPBA, HPM, and TMSPM with the initial molar ratios of 20:1:1:1, initiated by AIBN (1% of total molar quantities for all reactants) at 60 °C for 12 h under nitrogen, then precipitated and freeze-dried at −60 °C under vacuum for 24 h, and finally stored in a refrigerator for further use. The self-made bottom-spray fluidized bed reactor was designed and constructed for spray coating on glass microspheres. The synthesis of p(NIPAAm-*co*-AAPBA-*co*-HPM-*co*-TMSPM) copolymers, flow diagram of fluidized bed reactor and schematic diagram of copolymer microcapsule formation is shown in Figure 1.

P(NIPAAm-*co*-AAPBA-co-HPM-*co*-TMSPM) copolymers were dissolved in absolute ethanol at the concentration of 30 mg∙mL^−1^, stirred at room temperature for 12 h, and then purified with Millipore filters (0.2 μm) to remove any impurities. The copolymer solution was poured into the reservoir, and a moderate amount of glass microspheres were placed in the bottom-spray fluidized bed reactor, and kept in steady suspension by blowing. The atomizer was turned on immediately and turned off after 60 s, and ventilation was continued to accelerate the evaporation of absolute ethanol on the sphere surfaces for 120 s. The above spraying process was repeated 10 times to obtain higher wall thickness of the copolymer microcapsules. The operation parameters of the reactor system were described as follows: temperature of 20 ± 1 °C, supply air rate of 170 ± 5 m^3^/h, liquid spray rate of 1.7 ± 0.1 L/h, and atomization pressure of 0.2 MPa. Then the coated glass microspheres were put in a vacuum oven and annealed at 125 °C for 3 h. After natural cooling, the modified glass microspheres were soaked and washed in absolute ethanol and deionized water thoroughly to remove any unconnected copolymers, and collected by filtration and dried with vacuum drying at 20 °C. Finally, the prepared core–shell microspheres were dispersed in 20% HF solution. After soaking for 5 h, the copolymer microcapsules were separated by filtration, rinsed fully with deionized water, and further dried under vacuum for use.

The chemical composition of the copolymer microcapsules were measured by ATR-FTIR in the wavenumber range of 4000~500 cm^−1^. For equilibrium swelling measurements, the weight of 100 randomly selected dry core–shell microspheres were first determined (*W*_dry_), and then the modified microspheres were submerged into deionized water at 20 °C and 40 °C, PBS solution with pH 7.4 at 20 °C, as well as 5.0 mg∙mL^−1^ glucose solution with pH 7.4 at 20 °C and 40 °C for 24 h, respectively. The swelling behavior was evaluated by filtrating the soaked core–shell microspheres, and immediately measuring the weights, corresponding to *W*_*wet*,water,20°C_, *W*_*wet*,PBS,20°C_, *W*_*wet*,water,40°C_, *W*_*wet*,glucose,20°C_, and *W*_*wet*,glucose,40°C_. All the above data of mass were removed from the original weight of glass microspheres. The swelling ratio was defined as [6,48]:

(1) $$\mathrm{Swelling}\;\mathrm{ratio}\;(\%)=\frac{W_{wet}-W_{dry}}{W_{dry}}\times 100\%$$

The diameters of copolymer microcapsules under different conditions were measured via an electronic micrometer. The arithmetic mean of the microcapsule size was obtained from the measurements of 200 randomly selected microcapsules. The surface topography of copolymer microcapsules upon immersing into deionized water at 20 °C and 40 °C, PBS solution with pH 7.4 at 20 °C, as well as 5.0 mg∙mL^−1^ glucose solution with pH 7.4 at 20 °C and 40 °C were observed via SEM to determine the thermosensitivity and glucose-sensitivity. 

To investigate the stability of the copolymer microcapsules in different solution environments, the weight of fully dried microcapsules were first recorded, and then the microcapsules after immersion in deionized water, PBS solution (pH 7.4) and 5.0 mg∙mL^−1^ glucose solution for up to 28 days at 20 °C and 40 °C under sterile conditions separately and vacuum drying were weighted every 7 days. 

### 3.3. Encapsulation and Culture of β-TC6 Cells

The copolymer microcapsules with the average diameter of 500 μm were immersed in PBS solution at 20 °C until equilibrium swelling, and kept in a vacuum oven at 20 °C for 24 h. The fully dried copolymer microcapsules were soaked in 75% ethanol and sterilized by ultraviolet (UV) irradiation, and subsequently placed on a 24-well plate for further use. 

β-TC6 cells were seeded in culture flasks using HG-DMEM containing 10% FBS and 1% P/S as high-sugar culture medium (wherein the glucose concentration was 4.5 mg∙mL^−1^), and cultured at 37 °C in humid air with 5% CO_2_. The high-sugar medium was replaced every 3 days before cell encapsulation. The adherent β-TC6 cells were treated by 0.25% trypsin/0.02% EDTA. After centrifugation, the extracted cells were resuspended in sugar-free DMEM medium containing 10% FBS and 1% P/S. Ten microliters of cell suspension with the density of 5 × 10^4^/mL and the temperature of 4 °C was slowly injected into the dry sterilized copolymer microcapsules by microsyringe (VWR International, LLC) at 20 °C. The cell-microcapsule systems were diluted by 37 °C sugar-free culture medium and stood for 3 h at 37 °C. At this point, the pore size of the microcapsules suddenly decreased, and accordingly β-TC6 cells were trapped into the microcapsules. The centrifuged supernatant was used for counting unencapsulated cells, and accordingly the encapsulation efficiency of β-TC6 cells was calculated by following formula.

(2) $$\mathrm{Encapsulation}\;\mathrm{efficiency}=\frac{\mathrm{Total}\;\mathrm{number}\;\mathrm{of}\;\mathrm{seeded}\;\mathrm{cells}\;-\mathrm{Number}\;\mathrm{of}\;\mathrm{suspended}\;\mathrm{cells}}{\mathrm{Total}\;\mathrm{number}\;\mathrm{of}\;\mathrm{seeded}\;\mathrm{cells}}\times 100\%$$

After cell encapsulation, the hybrid systems of copolymer microcapsules and β-TC6 cells (i.e., biomicrocapsules) were suspended in high-sugar culture medium. The same amount of biomicrocapsules (groups of 100 microcapsules, each initially containing about 500 cells) were placed into 24-well cell culture plates, and incubated at 37 °C in humid air with 5% CO_2_. Then the biomicrocapsules were randomly selected at regular intervals from 0 to 192 h (i.e., 0~8 d) for the following tests. Two microliters of calcein-AM (excitation wavelength of 490 nm, emission wavelength of 515 nm) and 4 µM PI (excitation wavelength of 535 nm, emission wavelength of 617 nm) were added to PBS solution, which was employed as a working solution for cell dead/alive double staining. The biomicrocapsules were rinsed with PBS solution at 37 °C before adding 2 mL of working solution into each well, and then incubated at 37 °C for 15 min, followed by washing with PBS solution of 37 °C. The staining of cells was observed by inverted fluorescence microscope. The pictures of different colors in the same field were overlaid with Image-Pro plus 6.0 software (Media Cybernetics, Inc., Silver Spring, MD, USA). DOJINDO CCK-8 kit was employed to compare the growth and proliferation of β-TC6 cells with the same density under encapsulation and conventional models. 10 μL of CCK-8 solution was added to each well after the culture medium was replaced completely, and incubated for 3 h. The absorbance at 450 nm was measured with a colorimetric microplate reader. The absorbance was calculated as the cell number from working curve. The results were averaged from six repeated runs with standard deviation.

### 3.4. Glucose Responsive Insulin Secretion and Release of β-TC6 Cells

For insulin secretion experiments in vitro, the biomicrocapsules were suspended in LG-DMEM containing 10% FBS and 1% P/S as low-sugar culture medium (wherein the glucose concentration was 1.0 mg∙mL^−1^), and then placed into 24-well cell culture plates with the same amount (groups of 100 microcapsules, each containing nearly 500 cells) and incubated at 37 °C in humid air with 5% CO_2_ over a period of 28 days with replacing low-sugar culture medium every 3 days. At regular intervals (Day 0, 7, 14, 21 and 28), samples from six wells were taken and diluted with low-sugar culture medium of 37 °C, followed by incubating for 60 min at 37 °C [14,49]. The supernatants were collected at the end of the incubation period and analyzed for insulin content using insulin ELISA kit.

For evaluating glucose stimulated insulin secretion, the microencapsulated β-TC6 cells at the logarithmic growth phase with the approximately identical number of seeded cells (groups of 100 microcapsules, about 500 cells per microcapsule) were plated in 24-well culture plates. The biomicrocapsules were preincubated in sugar-free DMEM medium for 60 min, and were exposed to exogenous glucose with the final concentrations of 0~5.0 mg∙mL^−1^ in a total volume of 1 mL for 60 min. The supernatants were collected following glucose exposure to determine the insulin content of samples. Free β-TC6 cells with the same number cultured in 24-well culture plates were used as controls. The results were expressed as mean ± standard deviation of six independently parallel experiments.

In this study, insulin content was estimated by mouse insulin ELISA kit. The standard curve of insulin content with absorbance value could be determined by freeze-dried insulin extracted from mouse plasma (i.e., standard insulin). Ten microliters of culture supernatant from the samples was removed to 96-well ELISA plates, and supplied the standard dilution to 100 µL. Other steps were operated according to the instructions of ELISA kit. The absorbance was measured at 450 nm using a colorimetric microplate reader. The insulin concentration in the samples was calculated according to the standard curve and dilution ratio.

### 3.5. Bioactivity of Secreted and Released Insulin

The structural stability of secreted and released insulin derived from free and encapsulated β-TC6 cells was comparatively characterized by circular dichroism spectrometer and fluorescence spectrometer respectively. The secondary structure of produced insulin was measured by a circular dichroism spectrometer with the scanning wavelength ranging from 200 to 250 nm. The fluorescence emission spectra of generated insulin with the excitation wavelength fixed at 276 nm and the emission wavelength scanning range between 290 and 380 nm were compared to observe the conformation of insulin, so as to estimate the maintenance of biological activity of released insulin. 

### 3.6. Statistical Analysis

The experimental data were analyzed by OriginPro 8.5 software (OriginLab Corporation) and the results were expressed as mean ± standard deviation of at least three independent observations unless otherwise stated. The statistical comparisons were performed by Student’s *t*-test and *p* < 0.05 was considered to be statistically significant.

## 4. Conclusions

In this study, copolymer microcapsules prepared by combining bottom-spray coating technology with template method maintained the temperature and glucose dual-responsive characteristics of pristine p(NIPAAm-*co*-AAPBA-*co*-HPM-*co*-TMSPM) copolymers, and also had excellent stability. It was confirmed that the swelling ratio and pore size of the copolymer microcapsules increased with the increase in glucose concentration or the decrease in temperature. The insulinoma β-TC6 cells encapsulated in the copolymer microcapsules had better cellular growth and proliferation behaviors, and could produce insulin under glucose stimulation. The secreted and released insulin from encapsulated β-TC6 cells which retained the specific bioactivity. Owing to the limitation of microcapsule structure, the glucose concentration corresponding to the insulin release peak of encapsulated β-TC6 cells increased to more than 1.0 mg∙mL^−1^ compared with that of the free cells (0.5 mg∙mL^−1^). This pattern of insulin release is beneficial because a higher level of glucose is being consumed continuously while insulin is being released at peak levels, which is useful for the clinical treatment of diabetes. The intelligent drug delivery system in this study can simulate the behaviors of insulin secretion and release in the human body, providing new ideas and technical supports for the research and development of artificial pancreas.

## Data Availability

Not applicable.

## Sample Availability

Samples of the compounds p(*N*-isopropylacrylamide-*co*-3-acrylamidophenylboronic acid-*co*-hydroxypropyl methacrylate-*co*-3-trimethoxysilylpropyl methacrylate), p(NIPAAm-*co*-AAPBA-*co*-HPM-*co*-TMSPM) copolymers, core–shell microspheres, and microcapsules are available from the authors.
